# Black Pete through the Eyes of Dutch Children

**DOI:** 10.1371/journal.pone.0157511

**Published:** 2016-06-20

**Authors:** Judi Mesman, Sofie Janssen, Lenny van Rosmalen

**Affiliations:** Centre for Child and Family Studies, Leiden University, Leiden, the Netherlands; University of Jyväskylä, FINLAND

## Abstract

The traditional figure of Black Pete seen during the December festivities around Sinterklaas (the Dutch Santa Claus) in the Netherlands has sparked fierce debates about his racial stereotypical characteristics and his potentially negative effects on children’s opinions about black people. The Black Pete phenomenon has even been discussed by the United Nations Committee on the Elimination of Racial Discrimination, resulting in a report urging the Netherlands to eliminate this form of racial stereotyping. The adult debate about Black Pete is clearly important, but Sinterklaas is essentially a children’s holiday. Surprisingly, there have never been any systematic studies to examine children’s views on Black Pete. The current study is the first to do so. In a sample of 201 children aged 5–7 years, we collected free descriptions of Black Pete, asked children to group him in relation to other figures, and to assign characteristics to him and comparison figures. The results showed that (1) Children are clearly aware of Black Pete’s skin color and subordinate status; (2) Children associate Black Pete more with clowns than with black people; (3) Children evaluate Black Pete very positively, but the positive characteristics do not generalize to their evaluation of black people. The findings illustrate the deep-rooted childhood origins of many Dutch people’s affection for Black Pete and their lack of awareness of his relation to racial stereotypes. This explains the resistance to changing the Black Pete figure and the slowness of the change process on this front.

## Introduction

The Netherlands is famous for many things, some positive, some negative, some controversial. The Dutch celebration of Sinterklaas ranks among the most controversial. On the 5th of December, Sinterklaas is celebrated in the Netherlands and in parts of Belgium, and the former Dutch colonies (such as Surinam). Children receive gifts from the legendary bishop who has supposedly sailed with his steamer from Spain to Holland accompanied by his mostly male and sometimes female helpers who all go by the name of Zwarte Piet (Black Pete). Black Pete traditionally has his face painted black (much like a Blackface Minstrel), his lips red, wears an afro wig and gold hoop earrings, and dresses like a Moorish page from the 17th century. Their arrival in the Netherlands, a few weeks before the actual celebration, is broadcast on national television. The Dutch consider Sinterklaas the most important tradition in the Netherland [1].

The roots of this celebration go back a long way and although many Dutch find the idea of any change in the tradition unacceptable, the fact of the matter is that it has changed many times. The first signs of a Sinterklaas tradition, based on the catholic bishop Saint Nicholas who died in 342 AD, are found in the Middle Ages. For centuries, Sinterklaas operated on his own [2], riding the roofs on his white horse and throwing presents through chimneys, or punishing naughty children by spanking them or taking them with him in a bag. A white helper is first mentioned around 1800 [2], but in the 1820s he somehow changed color and became a "curly-haired negro" [3]. The first illustration of a black helper appeared in a children's book on Sinterklaas in 1850 and is generally considered the introduction of Black Pete as he is presently known [4]. Interestingly, even Sinterklaas himself has been described as black in some writings [3], [5].

Dutch folklore would have it that Black Pete is black because he enters the house through the chimney and this is what many parents tell their children. The fact that this does not explain the afro hair, red lips and clean clothes is easily overlooked by young children (and adults). Some scholars have suggested that Black Pete originated from German mythology [6], from rituals around Europe in which people blacken their faces to look like scary, devil-like creatures [7], [8]. Another line of inquiry focuses on Black Pete’s potential slavery origins, based on his similarities to black children in old paintings wearing similar outfits and a metal collar, referring to their slave status [9], or to children on paintings depicting a black page [2].

Black Pete has been the cause of much debate (e.g., [10], [11], [12]). People from a variety of ethnic backgrounds have started to oppose the submissive role and racially stereotypical looks of Black Pete, claiming this amounts to racism, and a negative portrayal of black people that teaches children racist ideas. A recent documentary aired online by CNN provides a clear picture of the Black Pete phenomenon, its origins, and how the character is perceived by black people [13]. A survey showed that the majority of the Dutch population do not agree with the view that Black Pete is a racist character and want to maintain the traditional Black Pete [14]. The debate is now strongly polarized, and has involved riots, police interventions, court cases, and angry polemics in the media. The Black Pete phenomenon has even been discussed by the United Nations Committee on the Elimination of Racial Discrimination, resulting in a report urging the Netherlands to eliminate this form of racial stereotyping [15].

The largest group of black minorities in the Netherlands consists of those originally from the former Dutch colonies of Surinam (Creole subgroup) and the Antilles, and together constitute about 3% of the Dutch population [16]. Their socioeconomic status (SES) is on average lower than that of the Dutch majority, but higher than the SES of minorities from Morocco and Turkey [17]. Then there is the black minority from African countries (excluding those from the Arabic countries in North Africa), estimated at about 1% of the Dutch population [16]. Their specific SES is difficult to summarize, because they are generally merged with other groups in the category ‘other’ in statistics on ethnic minorities. In terms of social status, the black minority tends to have a better reputation in the Netherlands than the Muslim minorities from Moroccan and Turkish descent [18]. However, part of this positive reputation can be seen as a form of racism, with stereotypes such as black people being good dancers, athletic, and sexually attractive. Further, surveys show that black minority adults and children in the Netherlands experience racial discrimination [19], [20], and experimental studies reveal that Surinamese people are discriminated against in job application procedures [21]. Thus, the figure of Black Pete exists in a cultural context of both positive and negative stereotypes about black people.

Surprisingly, children’s ideas about and attitudes towards Black Pete have never been researched, even though Sinterklaas is essentially a children’s party. We simply do not know how children perceive Black Pete, whether they associate him with black people, and whether these associations are negative in nature. Anecdotally many Dutch people can recount stories of children calling a black person Black Pete, either mistakenly (especially in small children) or intentionally (to hurt the person’s feelings), but to what extent this constitutes a common association is unclear. Studying children’s views on Black Pete can contribute to our understanding of both the origins of many Dutch people’s strong feelings about maintaining the traditional Black Pete, as well as the potential consequences of exposure to Black Pete on children’s evaluations of black people.

The current study aims to investigate (1) children’s awareness of Black Pete’s skin color and subordinate status; (2) their categorization of Black Pete as a fantasy figure or a black person or something else entirely; (3) their evaluations of Black Pete and whether these generalize to their evaluations of black people.

## Methods

### Participants and procedure

Families with children aged 5–7 years were recruited through Facebook and via the networks of 13 student assistants who were involved in the data collection. We chose the age range of 5–7 years as this is the group who still believes in the Sinterklaas story and are old enough to complete the tasks we designed. A total of 201 families participated. One of the parents filled in an online questionnaire about the family’s background characteristics (see Table 1). After obtaining informed consent from both parents, families were visited at home by a student assistant who administered a set of standardized tasks to the child. The study was conducted in the month preceding the nationwide broadcast of the arrival of Sinterklaas and his Black Petes. This time frame was chosen to represent a period in which Black Pete is a salient figure as children start anticipating the festivities, schools start introducing Sinterklaas activities and shops start to put Black Pete figures in their windows and on their merchandise. We chose to stop collecting data after the weekend of the arrival of Sinterklaas so as not to confound the data with a strong increase in Sinterklaas-related events and direct exposure to actual Black Pete figures. Informed consent on behalf of the children enrolled was written, i.e., both parents signed a consent form. These forms were all collected in a folder kept in a locked cabinet and recorded in a password-protected excel file. All study methods and procedures (including the consent procedures) were approved by the Ethics Committee of the Institute of Education and Child Studies at Leiden University.

**Table 1:** Sample characteristics.

*Mother as main respondent*	*94%*
*Two biological parents in the home*	*93%*
*Both parents high education*	*51%*
*Age target child*	
-*5 years*	*39%*
-*6 years*	*34%*
-*7 years*	*26%*
*Focus child identified by parent as white*	*82%*
*All family members identified by parent as white*	*75%*
*Predominantly white neighborhood (0–25% non-white)*	*77%*
High ethnic salience in daily life*	*31%*

* White family in non-white neighborhood or non-white family in white neighborhood)

### Measures

#### Who is Black Pete?

To obtain children’s free descriptions of Black Pete, the assistant asked the children the open question ‘Who is Black Pete?’. After the first answer (or if there was no answer), the assistant would prompt further information by asking more specifically: ‘what does he look like, what does he do?’. Children’s answers to the first general question (Who is Black Pete?) were categorized as follows: (1) Pete’s black skin color, when children mentioned the color black as an attribute of Black Pete’s appearance. This included the following statements: ‘he is black’, ‘his face is black’, ‘his skin is black’. Simply repeating the name Black Pete, or just the word ‘black’ was not coded in this category; (2) Other aspects of Pete’s appearance, such as his colorful clothes, his hat with the feather, or his black curly hair; (3) The fact that Pete brings presents; (4) The fact that Pete is Sinterklaas’ helper, or using words such as page, servant, or slave, or when they describe how Pete helps Sinterklaas and has to do what Sinterklaas tells him to do; (5) Other, which included a variety of answers, such as mentioning that Pete is nice, or climbs through the chimney, or does tricks.

#### Categorizing Black Pete

Children were first presented with a sorting task based on the work of Lam and colleagues [22], with 12 cards showing a picture of either a Black Pete (P), a clown (C), a black person (B), or a white person (W), with three cards per category. Children were then asked to sort the cards into two piles, putting together in one pile those who ‘belong together’. There were no restrictions regarding the number of cards on the two piles. A clown was chosen as a potential comparison figure because like Black Pete, clowns are also dressed up in colorful clothes, behave in a funny way, and their faces are also painted, but white rather than black. The figures in the pictures were taken from the internet (color photographs of real people), and were matched with regard to gender (boys got presented with the male versions, girls with the female versions) and all figures were shown full-length, wore colorful clothes, faced forward, smiled, and stood in regular non-clownish poses. The 12 cards were laid out on the table in three rows in a fixed order (row 1: CWCBP, row 2: PCWB, row 3: BPCW).

To familiarize the children with the nature of the task, they first sorted pictures of real animals (horses and pigs) and fake animals (rocking horses and piggy banks). More than three quarters of children (77%) sorted these according to animal type (horses versus pigs), and only 7% according to the real/fake dimension.

#### Labeling Black Pete

Children were also asked to assign 20 characteristics to the four types of figures. The assistant presented the children with four cups with a picture of either a Black Pete, a clown, a black person, or a white person. They were then asked to assign characteristics read out by the student assistant to two of the cups (‘which two do you think are < characteristic>?’). The characteristics were printed on little cards that the child could put in the cups. The characteristics included 8 positive labels (nice, friendly, cheerful, thinks of others, smart, important, hard worker, brave), 3 labels reflecting the Black Pete and potentially black person stereotype (dumb, lazy, helper), and 9 filler labels and negative attributes (e.g., naughty, scared, sad, mean, serious, funny, boss, thinks only of himself, not important). The characteristics were presented to the children in a fixed order, mixing positive, stereotypical, and filler characteristics. We computed a total positive label score by counting the number of positive labels that each figure had received (potential score range 0–8).

#### Parents’ opinions about Black Pete

Parents also filled in a questionnaire about their opinions on the Black Pete phenomenon as used in a nation-wide survey in 2014. Table 2 shows the results of the current sample. A new variable was computed to reflect the level of parents’ pro-change opinions about Black Pete, by counting the number of times parents replied in agreement with statements 1 through 10 presented in Table 2, yielding a potential and actual total score range of 0 to 10 (M = 3.42; SD = 3.39, and 27% with a score 0).

**Table 2:** Parents’ opinions about Black Pete.

	AGREE
*1*. *The discussion about Black Pete is justified*	*44%*
*2*. *Black Pete is a racist phenomenon*	*18%*
*3*. *Black Pete’s appearance should change*	*35%*
*4*. *It is justified that Amsterdam changed the appearance of Black Pete*	*34%*
*5*. *Big stores should change the appearance of Black Pete*	*29%*
*6*. *Toy brands should change their depiction of Black Pete*	*29%*
*7*. *Making Sinterklaas songs more neutral is a good initiative*	*40%*
*8*. *Even if unintentional*, *I understand that Black Pete comes across as discriminatory*	*53%*
*9*. *If a minority feels hurt by Black Pete*, *we should consider changing him*	*55%*
*10*. *Black Pete is a negative stereotype*	*19%*
*11*. *I have felt personally hurt by the racist nature of Black Pete phenomenon*	*5%*
*12*. *I tell my children that Black Pete is black because he comes through the chimney*	*81%*
*13*. *I tell my children that not everyone likes Black Pete*	*24%*
*14*. *I do not celebrate Sinterklaas with my children*	*0*.*5%*

Note: questions 1 through 10 also included the option ‘no opinion/don’t know’.

### Analyses

The main study variables are (1) whether or not children mentioned Black Pete’s skin color first in response to the open question; (2) whether or not children mentioned Black Pete’s skin color at all in response to the open question; (3) whether or not children categorized the four types of figures by skin color; (4) the number of positive labels children assigned to the four figures; (5) the number of times children assigned the same label to both Black Pete and the black person; (6) whether or not children assigned the stereotypical labels lazy, dumb, and helper to both Black Pete and the black person. The set of background variables tested in relation to the study variables are: child age, family SES, family skin color (all white versus not all white), ethnic salience (see Table 1), and parental pro-change opinions about Black Pete. For the analyses, family SES was computed as the sum of the standardized values of family income, maternal education, and paternal education.

To test the relations between the background variables and the test variables, several analyses were used depending on the nature of the variables. We conducted ANOVAs in case of a dichotomous study variable (almost all except for variables 4 and 5) and a continuous background variable (child age, family SES, parental pro-change opinions). If both study variable and background variable were dichotomous (e.g., first mentioning Pete’s skin color and family skin color), chi-squared analyses were conducted. In case of two continuous variables (e.g., number of positive labels and family SES) we conducted Pearson correlations. Finally, to compare the number of positive labels assigned to the four figures, a Repeated Measures ANOVA was conducted with within-subjects contrasts.

## Results

**Fig 1:**
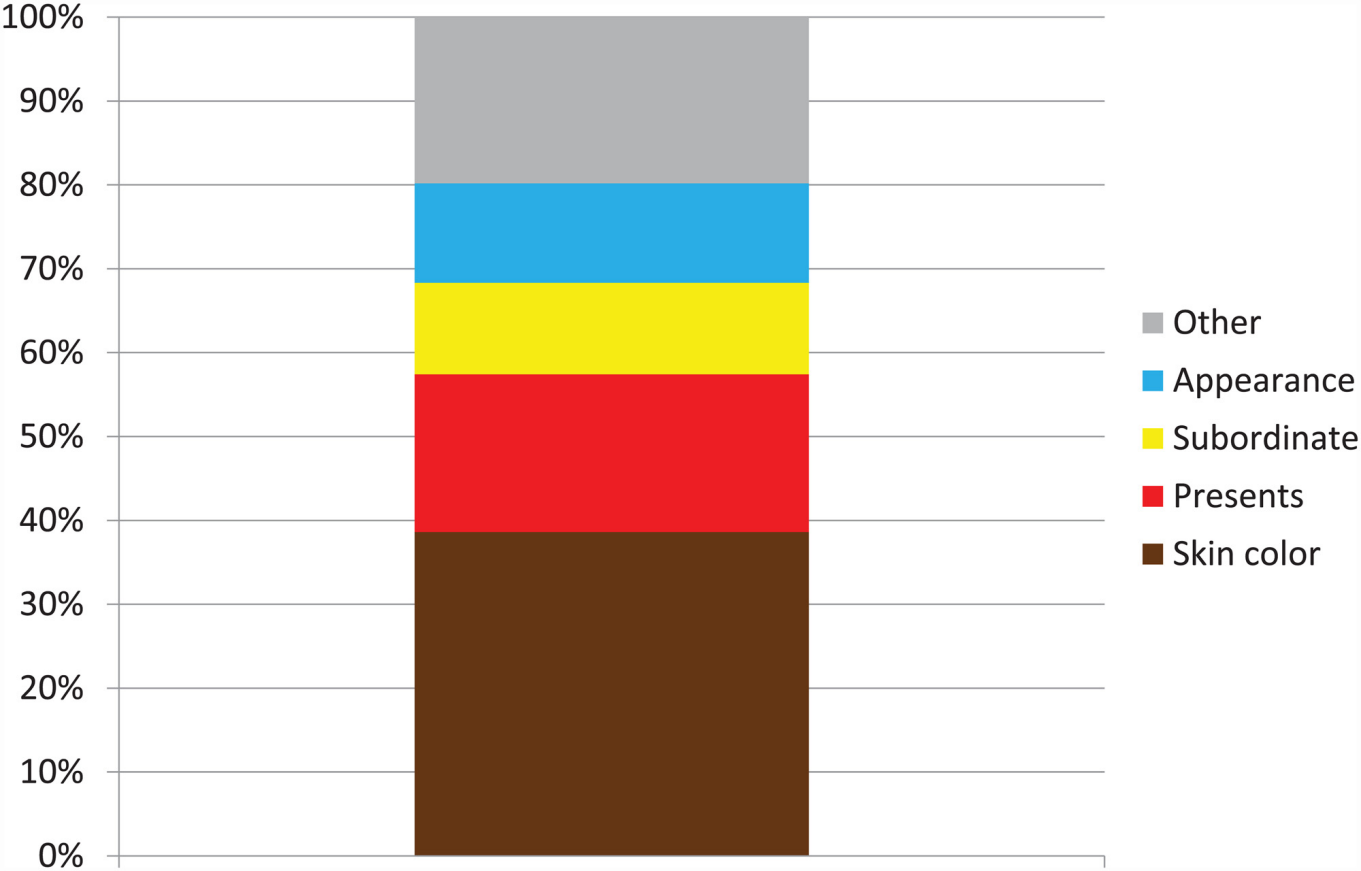
Children’s first answer to the question ‘Who is Black Pete?’

Fig 1 shows the children’s very first answer to the open question Who is Black Pete?, and reveals that the largest group of children first mentioned his skin color (39%). The fact that he brings presents was mentioned first by 19% of children, and his subordinate status of helper of Sinterklaas was the first answer provided by 11% of the children. When probed further about who Black Pete is, what he looks like, and what he does, the large majority of children mentioned his skin color (80%), his subordinate status (82%), and the fact that he brings presents (83%). No other characteristics were mentioned as often as these three. Two children did not answer the question at all. We found no relations between children’s or parents’ characteristics and children’s answers to the open question about Black Pete.

**Fig 2:**
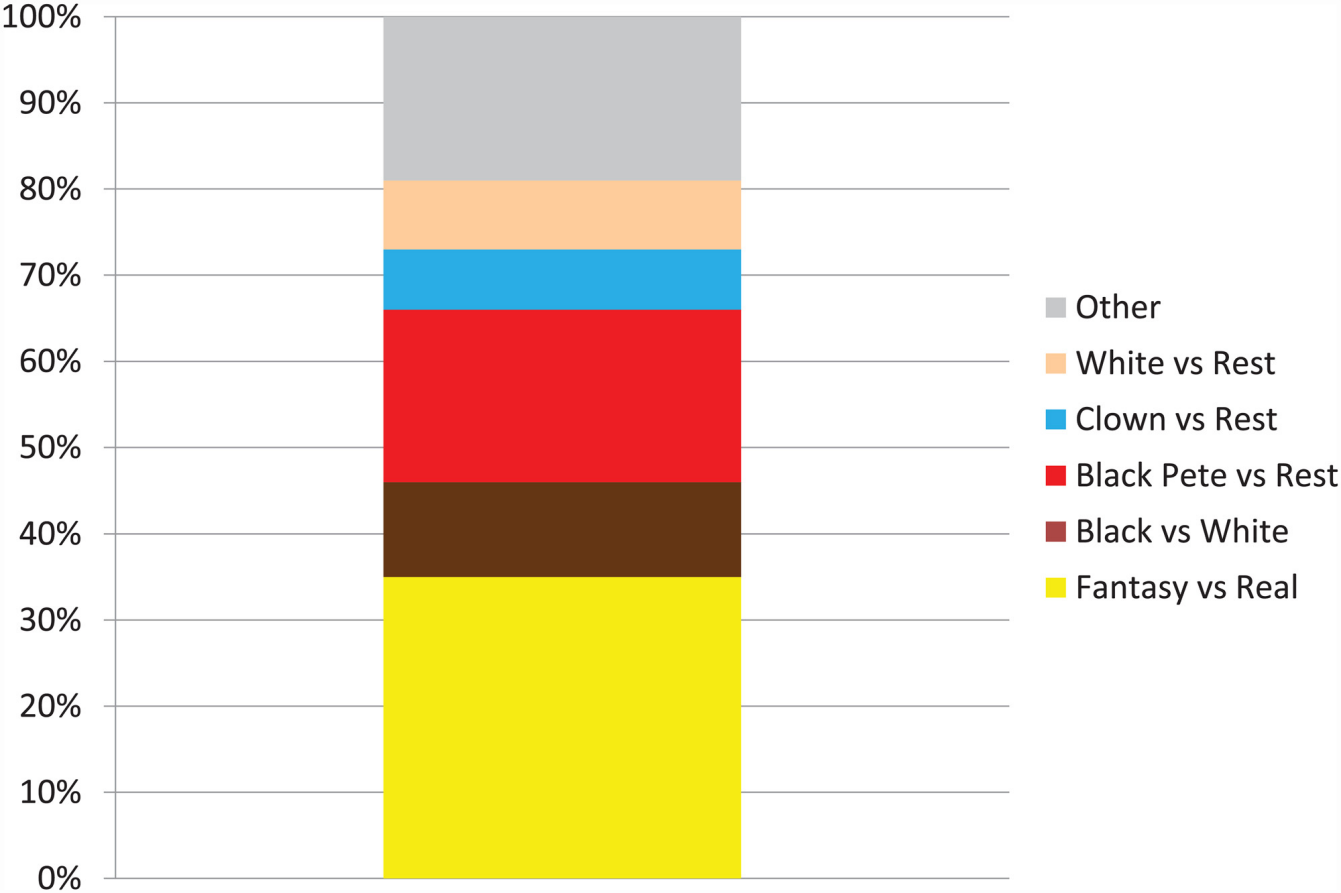
Children’s categorizations of Black Petes, clowns, black people, and white people.

The results of the card-sorting task are presented in Fig 2, and show that the largest group of children (35%) grouped the figures according to the fantasy versus real distinction, with Black Petes and clowns in one pile, and the white and black people in the other pile. Another 20% put the Black Petes into one pile and all other figures on another pile. Only 11% of the children grouped the figures according to skin color, putting Back Petes and black people together in one pile and clowns and white people in another pile. A substantial minority (19%) sorted the figures on completely different characteristics than identity or skin color, such as aspects of the clothing (e.g., those with versus without yellow clothing items, or those with and without their jackets open). Whereas some children sorted the figures as ‘white person versus the rest’ or as ‘clown versus the rest’, none sorted them as ‘black person versus the rest’.

**Fig 3:**
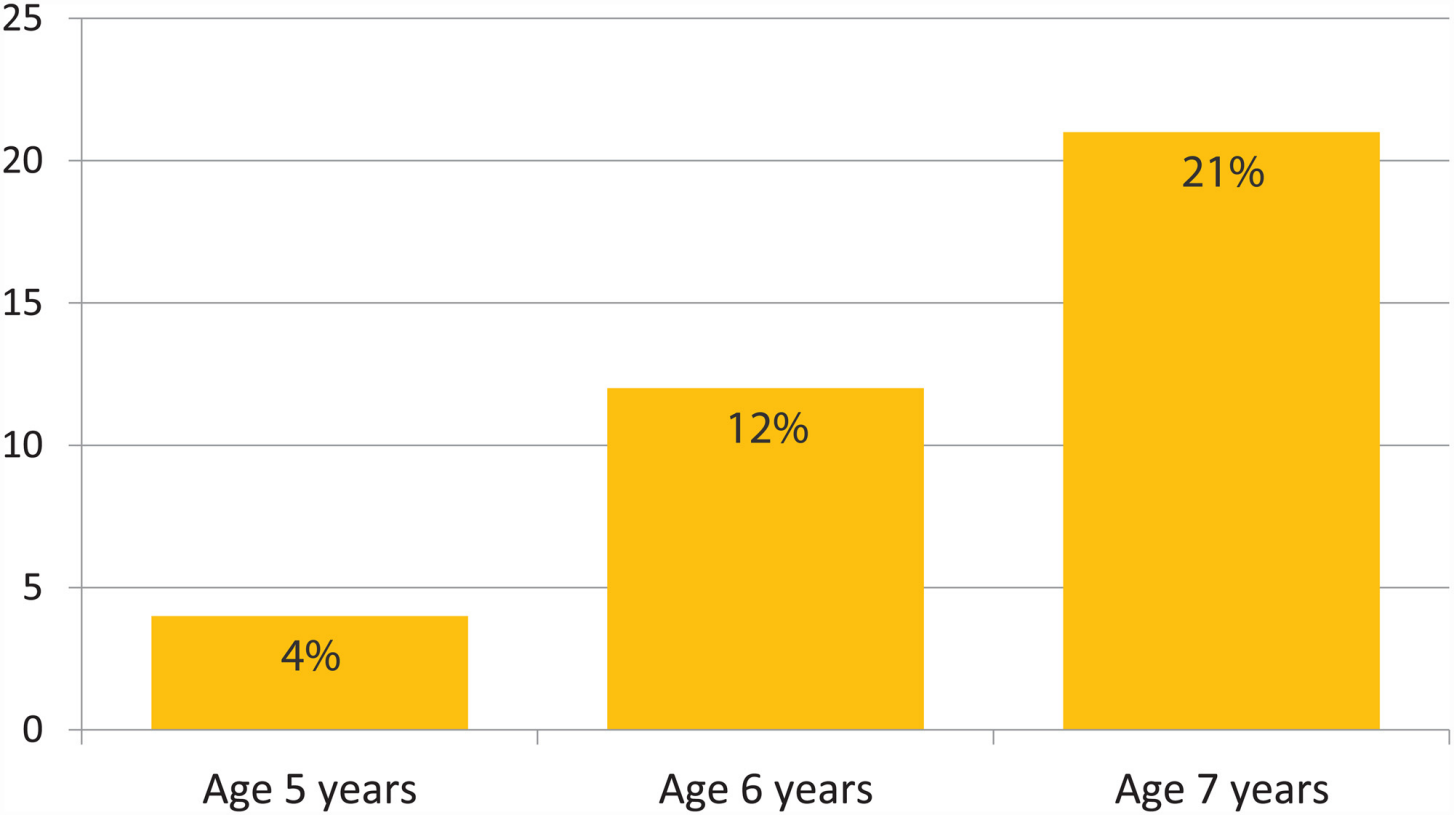
Categorizing by skin color across age groups.

We examined children’s responses to the card-sorting task in relation to background characteristics. Fig 3 shows that sorting by skin color increased significantly with age, χ^2^ (1, *N* = 201) = 9.40, *p* = .009. Only 4% of 5-year-olds sorted according to skin color, whereas this was the case for 12% of the 6-year-olds, and 21% of 7-year-olds. Further, sorting by skin color was more likely by children experiencing high ethnic salience in daily life (i.e., white families in non-white neighborhoods and vice versa) compared to other children, χ^2^ (1, *N* = 201) = 6.51, *p* = .011. Sorting by skin color was not related to other sociodemographic variables or the parent’s opinions about Black Pete.

**Fig 4:**
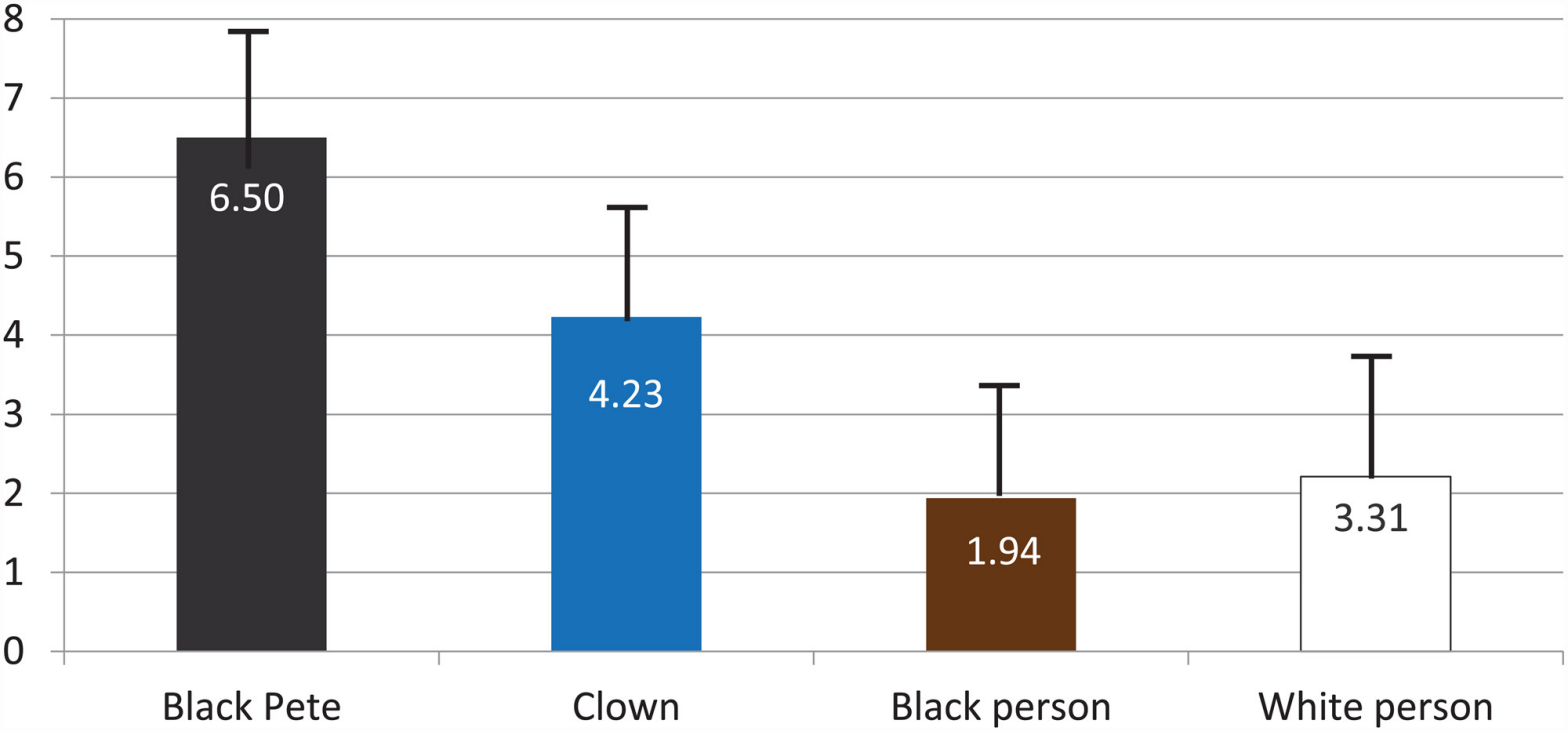
Mean number of positive labels (range 1–8) assigned to each character Black vertical lines represent standard errors.

Regarding children’s evaluations of Black Pete and the other figures (clowns, black people, white people), we found that positive labels were not equally distributed across the four figures, *Pillai’s F*(3, 198) = 118.31, *p* < .001, *η*_p_^2^ = .64. Fig 4 shows that children overwhelmingly assigned positive characteristics to Black Pete (average of 6.5 positive labels out of a possible 8), and significantly more so than to the other three figures. In fact, all within-subjects contrasts were significant at *p* < .001, indicating significant differences between each pair of figures, with the black person receiving the fewest positive labels. Older children assigned more positive labels to Black Pete, *F*(2, 198) = 14.11, *p* < .001, *η*_p_^2^ = .13, and fewer positive labels to the black person, *F*(2, 198) = 7.61, *p* = .001, *η*_p_^2^ = .07. No other relations between positive labeling and background variables were found.

**Fig 5:**
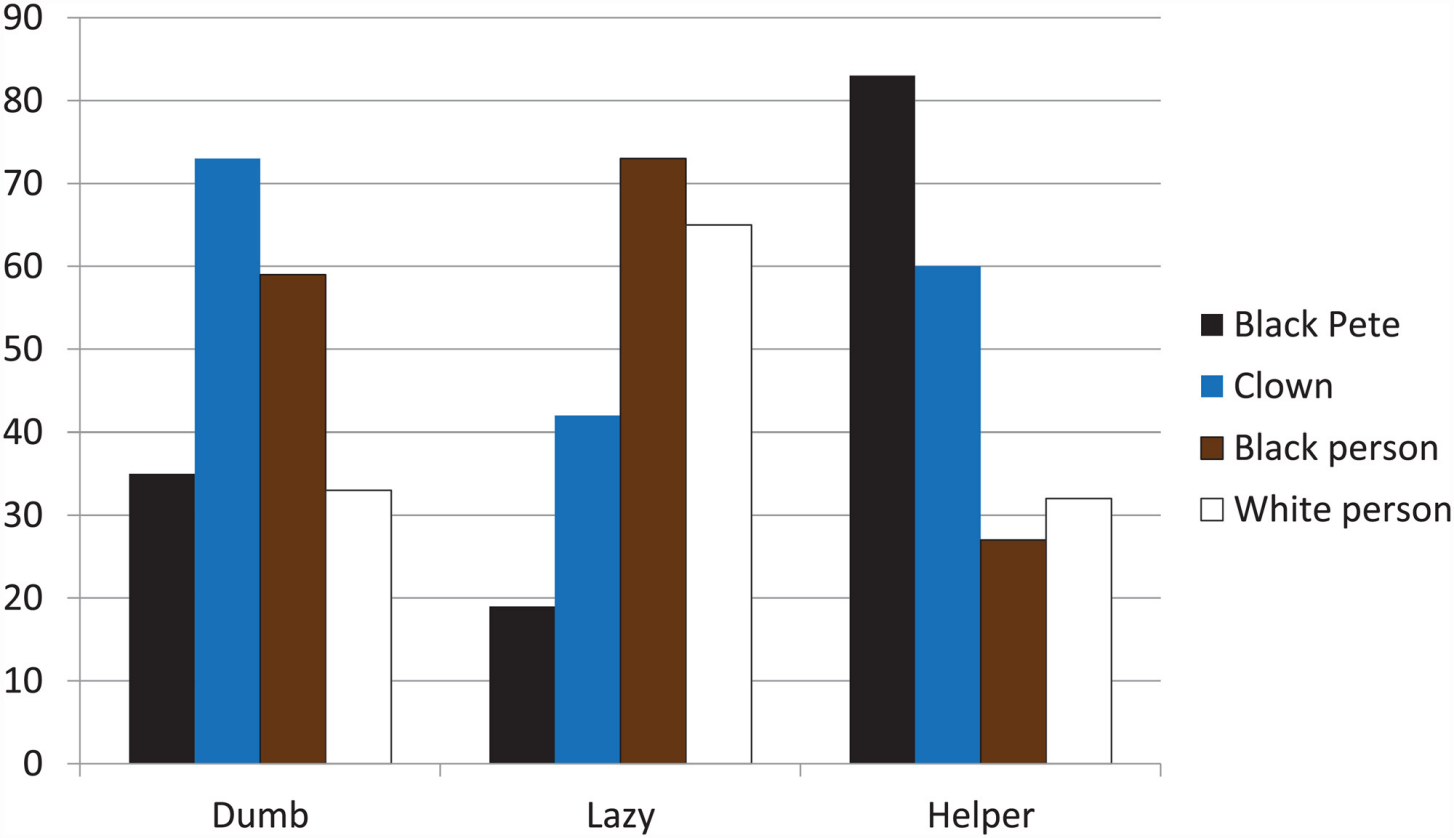
Percentages of stereotypic labels assigned to each character Because each label was assigned twice, chance distribution would yield 50% for each character.

We also looked specifically at the labels related to the stereotype of Black Pete and potentially of black people, including the characteristics dumb, lazy, and helper. Remember that the chance occurrence of receiving a label was 50%. Fig 5 shows that children do not see Black Pete as being dumb (35%) or lazy (19%), but they do clearly identify him as a helper (83%), and that these proportions are significantly different from 50% (*p* < .001). What is also clear is that children’s perceptions of Black Pete’s helper status do not generalize to their labeling of the black person who actually received the lowest number of ‘helper’ labels. Instead, children chose the clown for this label. The clown and the black person were overrepresented for the label Dumb, and the black person and the white person were overrepresented for the label Lazy.

Regarding associations between figures, we examined those instances when children assigned the same labels to Black Pete and the black person. This combination was made by 2–15% of children depending on the specific characteristic (noting that there were 6 possible combinations of figures, which means a chance rate of 17% for each combination). The stereotypical labels were much more often attributed to the Black Pete-clown combination than to the Black Pete—black person combination: helper (54% vs 11%), dumb (26% vs 7%), and lazy (11% vs 4%). The most prevalent Black Pete—black person combinations were those for the labels smart (15%), brave (14%), and thinks of others (11%).

We further examined whether some children were more likely to assign the same labels to Black Pete and the black person than other children were. We found that this combination was made more frequently by children of parents with more pro-change opinions about Black Pete *r*(199) = .15, *p* = .03, and more frequently by children from all-white families (M = 1.72, SD = 2.16) than by children from other families (M = 0.98, SD = 1.08), *F*(1, 199) = 5.32, *p* = .02, *η*_p_^2^ = .03. Similarly, the specific combination Black Pete—black person for the label Helper was also more commonly made by children from all-white families (14% made this combination) than by other children (2%), χ^2^ (1, *N* = 201) = 5.46, *p* = .03, but this was not found for the other stereotypical characteristics (lazy and dumb). There were no other relations between children’s pairing of Black Pete and the black person and any of the other background characteristics.

## Discussion

The results of the current study regarding Dutch young children’s perceptions of Black Pete can be summarized as follows: (1) Children are clearly aware of Black Pete’s skin color and subordinate status; (2) Children associate Black Pete more with clowns than with black people; (3) Children evaluate Black Pete very positively, but the positive characteristics do not generalize to their evaluation of black people. Some of these findings were partly dependent on participants’ background characteristics, but never on parental opinions about Black Pete.

Our first finding that children are aware of Black Pete being black (83% mentioned this when asked about Black Pete) is consistent with research findings showing that children already distinguish between people with different skin colors in the first year of life [23]. Thus, children are not color blind, as some in favor of keeping the traditional Black Pete have argued. The highly popular daily Sinterklaas television news broadcasts in the weeks preceding December 5^th^ have actually stopped calling Black Pete ‘black’ since several years now, and just call him Pete. However, the broadcasts still include mostly traditional black-faced Petes, and apparently just removing the label ‘black’ does not make children forget about his skin color.

Further, our results showed that children are also acutely aware of Black Pete’s status as a subordinate to Sinterklaas. Some children actually used the word ‘slave’ in their free descriptions of Black Pete, and most used the word helper or servant. This awareness of Black Pete’s subordinate status is not surprising given the storylines in the Sinterklaas news broadcasts where it is clear that the Black Petes have to do what Sinterklaas says and that they are responsible for all the hard work while Sinterklaas sits at his desk, sleeps in his bed, or rides on his horse. Sinterklaas can also frequently be seen to reprimand Black Petes for making mistakes in the news episodes. Also, many of the Sinterklaas songs refer to Black Pete as the helper or servant.

Interestingly, Black Pete being the helper of Sinterklaas does not diminish his status in the eyes of the children in our study. Black Pete is overrepresented as the receiver of positive labels such as smart, hard worker, important, and brave. This is probably due to the fact that Black Pete’s subordinate status goes together with him being assigned tasks that children find incredibly important, such as wrapping and delivering the presents and baking the seasonal candy goods. Black Pete was followed by the clown in terms of receiving positive evaluations from the children, then by the white person, then the black person. It appears that the two fantasy figures are very attractive to children and that regular people (white or black) can not compete with them. However, the black person also received significantly fewer positive labels than the white person, which is consistent with research from several countries showing that 3- to 7-year-old children (of any skin color) tend to prefer white people over black people [24]. Importantly, this pattern had never been studied in the Netherlands before, where the notion of children being color blind is still very popular.

We further found that children associate Black Pete more with clowns than with black people, both in the sorting task (where grouping by skin color was rare), and in the labeling task (where the Black Pete—black person combination of labels was rare). This is exactly what many Dutch people who want to keep the traditional Black Pete argue: children do not see Black Pete as a regular black person. In the public debate, adults maintain this position and often emphasize that Black Pete is only black because he enters the house through the chimney (no matter that his clothes stay clean). The effects of the color blindness that Dutch adults seem to propagate by negating Black Pete’s skin color as being relevant to his identity is particularly interesting in light of evidence that color-blind strategies actually increase racial tensions [25], which is actually exactly what is happening in the debate about Black Pete in the Netherlands.

Regarding the role of background variables, we found very few relations with the children’s responses to the tasks. Older children were much more likely to categorize by skin color (Black Pete and black person in one pile), and more likely to assign positive labels to Black Pete and fewer positive ones to black people. This is consistent with research showing that skin color becomes more salient in children’s evaluations of unknown persons as they get older [26]. Some effects of participant ethnicity were also found, with a higher likelihood of categorization by skin color in children experiencing more ethnic salience in daily life (i.e., white children in non-white neighborhoods and non-white children in white neighborhoods). Other studies have found similar effects, showing that experience with racial diversity enhances children’s awareness of race as a social category [27], emphasizing the importance of social context in understanding children’s racial attitudes as described by Developmental Intergroup Theory [28]. Finally, white children were more likely to make Black Pete—black person combinations in their evaluations in general, and regarding the label ‘helper’ in particular.

There were very few relations between the parents’ opinions about Black Pete and children’s responses to the tasks. This is likely to be due to the inherently secretive nature of the Sinterklaas festivities. Parents essentially lie to their children throughout the weeks leading up to December 5^th^, telling them that Black Pete will come through the chimney and put treats in their shoes, and that Sinterklaas writes their wishes in his big book. Thus, open conversations about Black Pete in which parental opinions are discussed and may influence children’s views are very unlikely when children are still ‘believers’. We did find one significant albeit weak effect of parental opinions, showing that children of more pro-change parents were more likely to make Black Pete—black person combinations in their evaluations. It may be that these children have overheard something about the debate and have therefore been exposed more to the notion that Black Pete may have something to do with black people.

Summarizing, the current study shows that young Dutch children see Black Pete as a positive figure, showing that he is unlikely to foster negative stereotypes about black people in Dutch children. However, positive stereotypes are also potentially harmful to the position of the stereotyped group as they tend to be linked to negative stereotypes and can be used to justify inequalities in treatment [29], [30]. Nevertheless, Fig 4 clearly shows that the positive attributes assigned to Black Pete do not generalize to children’s views of black people who received the lowest number of positive attributes. It might be possible that Dutch children actually contrast Black Pete and black people (as being comparable but different groups) leading to Black Pete receiving all the praise because of his fun look and being the bearer of gifts and candy, whereas black people not having any of these cool attributes are thus a less attractive versions of Black Pete. However, this speculation can not be directly inferred from our data and requires further investigation.

There are some limitations to the study. First, the study sample was not representative of the general Dutch population, with an overrepresentation of parents with a high educational level. The parents in this sample also had less conservative ideas about Black Pete than the general population (e.g., 44% of our sample finds the debate justified versus 19% of the general population, and 18% of our sample sees Black Pete as a racist phenomenon, versus 6% of the general population [14]. However, there were hardly any relations between parental opinions about Black Pete and children’s responses to the tasks, and no relations at all with family socioeconomic status, suggesting that a more representative sample would not have led to other conclusions regarding children’s lack of associations between Black Pete and black people.

A second limitation concerns the specific choice of comparison figures (clowns, black people, white people). As in any study that includes a certain set of figures or concepts that need to be compared or combined, the extent to which the results can be generalized beyond this specific set of figures is unclear [31]. Without the clown figure children might have been far more likely to group Black Pete with black people, although the fact that almost twice as many children chose to put Black Pete on a separate pile rather than putting him together with the black people suggests that the grouping by skin color is just not that prominent in this age group in relation to Black Pete. It is however important to consistently describe our results in relation to the studied figures (i.e., children associate Black Pete more with clowns than with black people).

A third limitation is the lack of a standardized Implicit Association Test (IAT). Although our card-sorting task and labeling task had implicit features, they also included more explicit aspects. Thus, some children may have responded in a socially desirable way, and avoided responses that could be interpreted as racist or discriminatory. Some studies have indeed found evidence of such processes [32], whereas others find that social desirability tendencies are unrelated to prejudice in children [33].

In conclusion, our results may give some insights into why the debate is so polarized and why many Dutch people are very unwilling to acknowledge the potentially racist characteristics of Black Pete and to allow the figure to evolve towards a less controversial appearance. We speculate that just like the children in this study, current Dutch adults have experienced Black Pete as a lovable figure from early childhood onwards, reminiscent of a clown rather than a real black person. As several studies have shown, being accused of racism (or in this case of admiring a figure that is seen as racist) predictably leads to anger at the messenger and resistance to the message [34], especially when the accused endorses color blindness [35]. It appears that this is the phase that the majority of the Dutch population is in. However, there is also evidence that anger and resistance are followed by change in attitudes and behaviors [26], suggesting that the message just takes time to sink in. Indeed, things are already changing. In 2015, one third of primary schools had already decided to change the appearance of Black Pete to avoid racial stereotyping. Many big commercial companies have also changed their Black Pete characters. It is most likely that the next generation of children’s will not know Black Pete anymore and that their parents will reminisce and say things like “I remember when Pete used to be black”. On the larger scale of things, the overhaul of a cherished cultural phenomenon within one generation is actually a very impressive feat.
